# Diosmin Protects against Ethanol-Induced Gastric Injury in Rats: Novel Anti-Ulcer Actions

**DOI:** 10.1371/journal.pone.0122417

**Published:** 2015-03-30

**Authors:** Hany H. Arab, Samir A. Salama, Hany A. Omar, El-Shaimaa A. Arafa, Ibrahim A. Maghrabi

**Affiliations:** 1 Department of Biochemistry, Faculty of Pharmacy, Cairo University, Cairo, 11562, Egypt; 2 Biochemistry Division and GTMR Unit, Department of Pharmacology and Toxicology, Faculty of Pharmacy, Taif University, Taif, 21974, Saudi Arabia; 3 Department of Biochemistry, Faculty of Pharmacy, Al-Azhar University, Cairo, 11751, Egypt; 4 Department of Pharmacology, Faculty of Pharmacy, Beni-Suef University, Beni-Suef, 62514, Egypt; 5 Sharjah Institute for Medical Research, College of Pharmacy, University of Sharjah, Sharjah, 27272, United Arab of Emirates; 6 Department of Clinical Pharmacy, Faculty of Pharmacy, Taif University, Taif, 21974, Saudi Arabia; University of Louisville, UNITED STATES

## Abstract

Alcohol consumption has been commonly associated with gastric mucosal lesions including gastric ulcer. Diosmin (DIO) is a natural citrus flavone with remarkable antioxidant and anti-inflammatory features that underlay its protection against cardiac, hepatic and renal injuries. However, its impact on gastric ulcer has not yet been elucidated. Thus, the current study aimed to investigate the potential protective effects of DIO against ethanol-induced gastric injury in rats. Pretreatment with DIO (100 mg/kg p.o.) attenuated the severity of ethanol gastric mucosal damage as evidenced by lowering of ulcer index (UI) scores, area of gastric lesions, histopathologic aberrations and leukocyte invasion. These actions were analogous to those exerted by the reference antiulcer sucralfate. DIO suppressed gastric inflammation by curbing of myeloperoxidase (MPO) and tumor necrosis factor-α (TNF-α) levels along with nuclear factor kappa B (NF-κB) p65 expression. It also augmented the anti-inflammatory interleukin-10 (IL-10) levels. Meanwhile, DIO halted gastric oxidative stress via inhibition of lipid peroxides with concomitant enhancement of glutathione (GSH), glutathione peroxidase (GPx) and the total antioxidant capacity (TAC). With respect to gastric mucosal apoptosis, DIO suppressed caspase-3 activity and cytochrome C (Cyt C) with enhancement of the anti-apoptotic B cell lymphoma-2 (Bcl-2) in favor of cell survival. These favorable actions were associated with upregulation of the gastric cytoprotective prostaglandin E_2_ (PGE_2_) and nitric oxide (NO). Together, these findings accentuate the gastroprotective actions of DIO in ethanol gastric injury which were mediated via concerted multi-pronged actions, including suppression of gastric inflammation, oxidative stress and apoptosis besides boosting of the antioxidant and the cytoprotective defenses.

## Introduction

Alcohol consumption has been commonly linked to gastric mucosal lesions including gastritis, gastric ulcer and even gastric carcinoma [1]. The mechanisms underlying ethanol-induced gastric ulcer have not been fully defined. Yet, mounting evidence has indicated that proinflammatory cytokines, oxidative stress and apoptosis play crucial roles in its pathogenesis [2–6]. Invasion of gastric tissues by neutrophils, marked with increased myeloperoxidase (MPO) activity, contributes to gastric mucosal damage [7]. Activation of neutrophils is associated with an upregulated inflammatory response with increased gastric expression of nuclear factor kappa B (NF-κB) which controls the generation of proinflammatory cytokines including tumor necrosis factor-α (TNF-α). These events amplify the inflammatory cascade via triggering the release of other proinflammatory mediators and enhancing further recruitment of macrophages and neutrophils, thereby exacerbating the gastric insult [6, 8]. Diminished gastric levels of the anti-inflammatory interleukin-10 (IL-10) have been reported in ethanol-induced mucosal injury [9, 10]. Meanwhile, oxidative stress has been implicated in the development of ethanol-induced gastric injury where an arsenal of reactive oxygen species (ROS) generated by activated leukocytes triggers mucosal damage via lipid peroxidation and via depletion of the antioxidant defenses such as reduced glutathione (GSH), glutathione peroxidase (GPx) and the total antioxidant capacity (TAC) [5, 9]. In the same context, enhancement of apoptosis and associated loss of mucosal epithelial cells are recognized as major players in the pathogenesis of ethanol-evoked gastric damage [2, 3]. Additionally, depletion of mucosal cytoprotective moieties, including prostaglandin E_2_ (PGE_2_) and nitric oxide (NO), has been intimately linked to ethanol consumption [9, 11].

The experimental model of ethanol-induced gastric injury mimics several features of the human condition and thus provides a mean for assessing agents with potential anti-ulcer actions along with their implicated mechanisms for gastric protection [9, 10, 12]. In the clinical setting, the current approach for the management of gastric ulcers focuses on the use of proton pump inhibitors and H_2_ receptor antagonists as the mainstay treatment. However, administration of these drugs has been associated with several adverse effects such as nausea, constipation, gynecomastia and impotence that limit their use [13]. Thus, the search for effective agents with fewer side effects has been regarded as an effective strategy for the management of gastric ulcers [5, 13, 14]. Previously, agents with antioxidant features have displayed beneficial actions in protecting against alcohol-induced gastric ulcer [4, 5, 9, 14]. Flavonoids are natural polyphenols that can protect human body against ROS-evoked damage and their consumption has potential health benefits including protection against various ailments [6, 15]. Diosmin, DIO, (diosmetin 7-O-rutinoside), a natural citrus flavone, has displayed remarkable antioxidant, anti-inflammatory, and anti-apoptotic activities in human and experimental models [15–20]. It is well known for boosting venous tone and lymphatic drainage with suppression of capillary hyperpermeability [21]. Interestingly, DIO has exerted versatile beneficial effects against experimental diabetes mellitus [18], myocardial infarction [17], hepatic and renal injuries [15, 19] along with hepatocarcinogenesis [20]. Yet, its impact on gastric ulcer has not been previously explored. Thus, the present study aimed to investigate the potential protective actions of DIO in ethanol-induced gastric injury in rats. Since the favorable actions of DIO are mainly ascribed to its antioxidant features, sucralfate was selected as the reference antiulcer agent in the current study due to its marked antiulcer/antioxidant properties [22–24]. Previously, sucralfate has displayed effective relief for the gastric ulcer symptoms in several experimental and clinical settings [24–26]. These beneficial actions are mediated, at least partly, via its antioxidant actions and through enhancement of gastric cellular antioxidant defenses [22–24]. Meanwhile, sucralfate inhibits proinflammatory cytokines and enhances the release of cytoprotective agents such as mucus and PGE_2_ [25, 27, 28].

## Material and Methods

### Ethics Statement

All animal handling and procedures were performed according to the Guide for the Care and Use of Laboratory Animals published by the US National Institute of Health (NIH publication No. 85–23, revised 1996). The current protocol was approved by the Committee of Animal Care and Use of Faculty of Pharmacy, Cairo University. All efforts were exerted in order to reduce the suffering of experimental animals.

### Animals

Adult male Wistar rats weighing 180–220 g were purchased from the National Institute for Research, Cairo, Egypt. The animals were maintained at controlled laboratory conditions (temperature (23±1°C), humidity (60±10%), and a 12/12 h light/dark cycle). Animals were allowed 1 week for acclimatization before any experimental procedures and they had free access to standard rat chow and water.

### Chemicals

Diosmin was purchased from Sigma-Aldrich, St. Louis, MD, USA whereas sucralfate was a kind gift from the Egyptian international pharmaceutical industries company (EIPICO), Egypt. All remaining chemicals used in the current study were of the highest quality and analytical grade.

### Induction of gastric ulcer

Gastric mucosal injury was induced using a single intragastric dose of absolute ethanol (5ml/kg) that was administered via orogastric intubation as previously described [2, 5, 9, 29]. The control group received the same volume of saline instead of ethanol.

### Experimental design and treatment protocol

Rats were placed individually in metabolic cages with raised floors of wide mesh in order to avoid coprophagy that interferes with the induction of gastric ulcer. The animals were fasted for 24 hours with free access to water. Then, 1 h before ethanol administration, the water was removed. Fourty rats were randomly assigned into five groups (8 rats per group). Group I (Control gp): normal rats which received oral vehicle (0.5% carboxymethyl cellulose; 5ml/kg). Group II (Control + DIO gp): normal rats that received oral diosmin (100 mg/kg/day) for 1 week. Group III (Ethanol gp): received a single intragastric dose of absolute ethanol (5ml/kg) + oral vehicle (0.5% carboxymethyl cellulose) for 1 week and the last dose of the vehicle was administered 1 h before ulcer induction. Group IV (Ethanol + DIO): received a single intragastric dose of ethanol + oral diosmin (100 mg/kg/day) for 1 week and the last dose of diosmin was administered 1 h before ulcer induction. Group V (Ethanol + SUCR): received a single intragastric dose of ethanol + an oral sucralfate (SUCR) dose (100 mg/kg) 1h before ulcer induction. DIO was prepared by suspension in 0.5% carboxymethyl cellulose vehicle and was daily administered by oral gavage. Animals were euthanized 1 h after ethanol and their stomachs were immediately dissected.

The selected dose of DIO was based on preliminary experiments and previous literature which demonstrated that 100 mg/kg of DIO displayed marked ameliorative and anti-inflammatory actions in animal models of diabetes [18, 30] and hypertension [31]. The dose of sucralfate (100 mg/kg p.o.), the reference anti-ulcer drug, is in accordance with previous reports [22, 24]. The chosen regimen is consistent with previous literature [4, 9, 32].

### Tissue collection and preparation

The animals were euthanized under deep ether anesthesia 1 hour post ethanol instillation. Following immediate laparatomy, stomachs were excised, opened along the greater curvature and rinsed with normal saline to remove gastric contents and blood clots. Stomachs were blotted dry, macroscopically inspected for gross gastric injury (expressed as ulcer index) and photographed for later determination of the area of gastric lesions. Then, each stomach was dichotomized, one moiety was immersed in 10% buffered formol saline for histopathologic and immunohistochemical assessment of NF-κB while the glandular gastric tissue of the other moiety was divided into 3 parts and was stored at -80°C for the biochemical investigations. To this end, one part was homogenized in 10 volumes of lysis buffer (200mMNaCl, 5mM EDTA, 10 mM Tris, 10% glycerine, 1 mM PMSF, 1 mg/ml leupeptin and 28 mg/ml aprotinin, pH 7.4) for the estimation of MPO, TNF-α, IL-10, caspase-3, Cyt C and Bcl-2. The second part was homogenized in 10 volumes of ice-cold phosphate buffer (100 mM, pH 7.4) for determination of oxidative stress markers and cellular defenses (MDA, NO, GSH, GPx and TAC). The third part was homogenized in phosphate buffer (10 mM, pH 7.4) containing 0.1 M indomethacin for estimation of PGE_2_.

### Determination of gastric ulcer index (UI) and percentage of inhibition

An observer who was blinded to the identity of samples examined the gastric tissues for gastric mucosal lesions which were expressed as UI. The degree of gastric mucosal damage was evaluated for gross pathology according to a 0–5 scoring system based on the number and severity of gastric lesions as previously described [33]: 0 = no lesions; 1 = small round hemorrhagic lesions; 2 = lesions <2 mm; 3 = lesions 2–3 mm; 4 = lesions 3–4 mm; 5 = lesion > 4 mm. The score was multiplied by 2 when the width of the erosion is larger than 1mm. The mean score was calculated and expressed as the UI. The percentage of inhibition was calculated by the following formula: [(UI _ethanol control_—UI _treated_)/UI _ethanol control_] × 100.

### Area of gastric hemorrhagic ulcerative lesions

The extent of gastric damage was quantified via measuring the area of gastric lesions. Following the dissection and opening of the rat’s stomach, it was thoroughly rinsed with cold saline to remove any contents or blood clots. Then, the flattened stomach samples were photographed. The digital photos were used for determination of the area (mm^2^) of the gastric lesions using imageJ 1.48d software (National Institute of Health, USA). For each stomach, the sum of areas of all forms of gastric lesions was recorded including the multifocal erosions and the linear hemorrhagic lesions.

### Histopathologic examination and microscopic scoring of gastric damage

Gastric biopsies were fixed in 10% buffered formol saline for 24h. Tissue samples were washed, dehydrated by alcohol, cleared in xylene and embedded in paraffin in hot air oven (56°C) for another 24 h. The paraffin blocks were cut into sections of 5 μm thickness and were stained with hematoxylin and eosin (H&E) and examined under the light microscope (Leica Microsystems, Germany). A qualified observer unaware of the identity of the specimens performed all the histopathologic procedures in order to avoid any bias. Gastric microscopic damage was scored on a 0–14 scale according to the criteria described by Laine and Weinstein [34]. Briefly, a 1 cm segment of each histological section was examined for epithelial cell loss (score: 0–3), edema in the upper mucosa (score: 0–4), hemorrhagic damage (score: 0–4), and the presence of inflammatory cells (score: 0–3).

### Immunohistochemical detection of NF-κB p65

For the antigen retrieval, paraffin-embedded tissue samples of 5 μm thickness were rehydrated in xylene and then in graded ethanol solutions and heated in citrate buffer (pH 6) for 5 min. Blocking was done using 5% bovine serum albumin (BSA) in Tris buffered saline (TBS) for 2 h. The samples were then incubated with primary polyclonal rabbit anti-NF-κB p65 (Santa Cruz Biotechnology Inc, CA, USA) at a concentration of 1 μg/ml in 5% BSA in TBS overnight at 4°C. The slides were then washed with TBS and were incubated with secondary goat anti-rabbit IgG using Vector Elite ABC kit (Vector Laboratories, Burlingame, CA, USA). Finally, the sections were washed with TBS and the immunoreaction was visualized using 3,3’-diaminobenzidine tetrahydrochloride (DAB Substrate Kit, Vector Laboratories Inc). Hematoxylin was used for counter staining and the slides were examined under a light microscope (Leica Microsystems, Germany) by a blinded observer.

### Gastric MPO activity and inflammatory cytokines (TNF-α and IL-10)

Myeloperoxidase (MPO) activity, a marker for neutrophil infiltration, was estimated by HK105 kit (Hycult Biotech, Uden, Netherlands) whereas the levels of TNF-α and IL-10 were determined by R &D systems ELISA kits (MN, USA) as instructed by the manufacturer. MPO as well as the cytokines assays employ quantitative sandwich enzyme immunoassay technique with specific antibodies pre-coated onto the microplate. The standards, control, and samples were pipetted into the wells and the rat cytokines were bound by the immobilized antibody. After washing away any unbound substances, an enzyme-linked secondary antibody specific for rat MPO, TNF-α or IL-10 was added to the wells. Following color development, the assay was stopped, and the absorbance was read at 450 nm. The intensity of the color was proportional to the amount of the corresponding target bound in the initial step. Results for MPO were expressed as (U/g tissue) whereas the cytokine levels were presented as pg/g tissue.

### Lipid peroxides concentration

The determination of lipid peroxides, expressed as malondialdehyde (MDA), was carried out according to the thiobarbituric acid assay [35]. The reaction proceeded in 15% w/v trichloroacetic acid, 0.375% w/v thiobarbituric acid and 0.25 N HCl at 90°C for 30 min. After cooling, the precipitate was removed by centrifugation and absorbance was recorded at 535 nm. The results were presented as nmol/g tissue.

### GSH levels

Gastric GSH levels were determined as previously described [36], where gastric proteins were precipitated with 10% trichloroacetic acid and the colored product was developed by 10 mM DTNB (5,5’- dithiobis 2-nitrobenzoic acid) solution in 0.3 M phosphate solution. The absorbance was measured at 412 nm and results were expressed as nmol/g tissue.

### GPx activity

Gastric GPx activity was determined using Sigma-Aldrich assay kit (Sigma-Aldrich, St. Louis, MD, USA) as instructed by the manufacturer. The kit utilizes an indirect determination based on oxidation of GSH by GPx which is then coupled by recycling the oxidized GSSG via glutathione reductase and NADPH. The decline in NADPH absorbance was monitored at 340 nm. One unit of enzyme is defined as the amount of enzyme that oxidizes 1 μmol of NADPH per min at 25°C. The protein content of gastric homogenates was determined using the method of Lowry et al. [37].

### Determination of TAC

The TAC was determined using Cayman total antioxidant assay kit according to the manufacturer’s suggestions. The capacity of gastric antioxidants to inhibit metmyoglobin-induced ABTS (2,2-azino-di-[3-ethylbenzthiazoline sulphonate]) oxidation was compared to Trolox, a water-soluble tocopherol analogue. The amount of oxidized products was measured at 405 nm and TAC was expressed as μmol of Trolox equivalent/g tissue.

### Caspase-3 activity

Caspase-3 activity was colorimetrically determined using R &D systems kit as instructed by the manufacturer. Caspase-3 cleaves the labeled substrate DEVD-pNA (acetyl-Asp-Glu-Val-Asp p-nitroanilide) releasing the chromophore pNA which was measured at 405 nm (Biochrom Asys microplate reader, UK). Results were expressed as fold change of caspase-3 activity.

### Cytochrome C (Cyt C) and B cell lymphoma-2 (Bcl-2)

Commercially available ELISA kits were used for the determination of gastric Cyt C (EIab ELISA kit) and Bcl-2 (Calbiochem, Darmastadt, Germany) as described by the manufacturer. The assays rely on a quantitative sandwich enzyme immunoassay technique and the final colored product was measured at 450 nm. The results for Cyt C were expressed as μg/ g tissue while those of Bcl-2 were presented as ng/g tissue.

### Estimation of PGE_2_

Determination of PGE_2_ was performed using corresponding ELISA kit (R &D systems incorporation, USA), as described by the manufacturer. The optical densities were measured at 450 nm and results were expressed as ng/g tissue.

### NO concentration

The levels of NO were determined as total nitrate/nitrite using Greiss reagent as previously described [38] with the modification of replacing zinc sulfate instead of ethanol for the precipitation of proteins in the homogenate supernatant [39]. Vanadium trichloride (in 1 M HCl) was added for the reduction of nitrate to nitrite, followed by rapid addition of Griess reagent consisting of N-(1-naphthyl) ethylenediamine dihydrochloride and sulfanilamide (in 5% HCl). The mixture was incubated at 37°C, allowed to cool and then the absorbance was measured at 540 nm. Results were expressed as nmol/g tissue.

### Statistical analysis

The data were presented as mean ± SEM, and the statistical analysis was performed using one-way analysis of variance (ANOVA), followed by Tukey-Kramer post hoc multiple comparisons test. Non-parametric values were presented as median and the statistical differences among groups were calculated using Kruskal-Wallis analysis of variance followed by the rank-based Mann–Whitney U-test. The analysis was performed using SPSS program, version 17. Differences were considered significant at p < 0.05.

## Results

### Diosmin pretreatment protects against ethanol-induced gastric injury in rats

**Fig 1:**
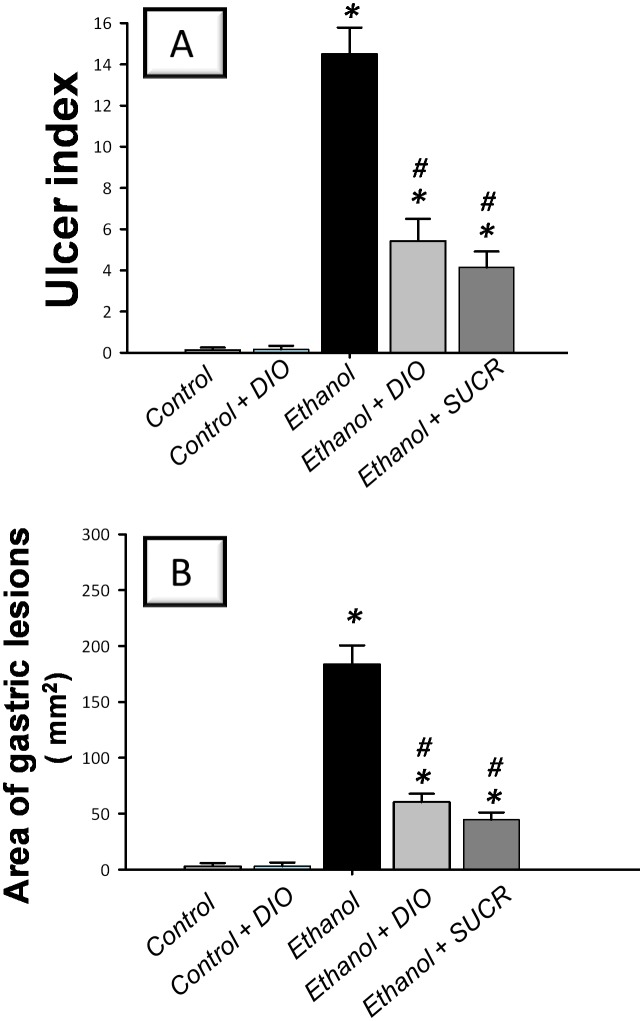
Diosmin lowers ulcer index (A) and area of gastric lesions (B) in rats with ethanol-induced gastric injury. Gastric ulcer was induced by a single intragastric administration of absolute ethanol (5ml/kg) whereas the control group received the same volume of physiological saline. Diosmin was orally administered (100 mg/kg/day p.o.), starting 1 week before ulcer induction while sucralfate (100 mg/kg p.o.) was administered 1 hr before ulcer induction. One hour post ethanol instillation, rats were euthanized and their stomachs were immediately excised. Scores of the ulcer index (non-parametric) are expressed as median; n = 6–8. *Significant difference from control gp at *p < 0*.*05*, *#* Significant difference from ethanol gp at *p < 0*.*05*. DIO; diosmin, SUCR; sucralfate.

To explore the severity of gastric injury, the signs of macroscopic damage were examined. Intragastric administration of absolute ethanol (5ml/kg; 1 h exposure) triggered severe hemorrhagic gastritis with high UI scores indicating several linear hemorrhagic ulcers and multifocal erosions compared to the vehicle-treated control group (Fig 1A). Meanwhile, large areas of hemorrhagic ulcerative gastric lesions were observed in ethanol-treated group revealing an extensive damage to the gastric mucosa compared to the control group (Fig 1B). Interestingly, pretreatment with DIO for 1 week significantly mitigated the gross gastric mucosal damage (63% inhibition of UI scores) and diminished the area of gastric lesions (67% inhibition) compared to ethanol group. Meanwhile, the reference anti-ulcer sucralfate afforded remarkable gastric protection against ethanol insult as evidenced by lowering of UI scores and ulcerative area by 71% and 76%, respectively, as compared to ethanol group. These data suggest that DIO attenuated the development of ethanol-induced gastric ulcerative lesions.

### Diosmin attenuates gastric histopathologic aberrations and influx of leukocytes

**Fig 2:**
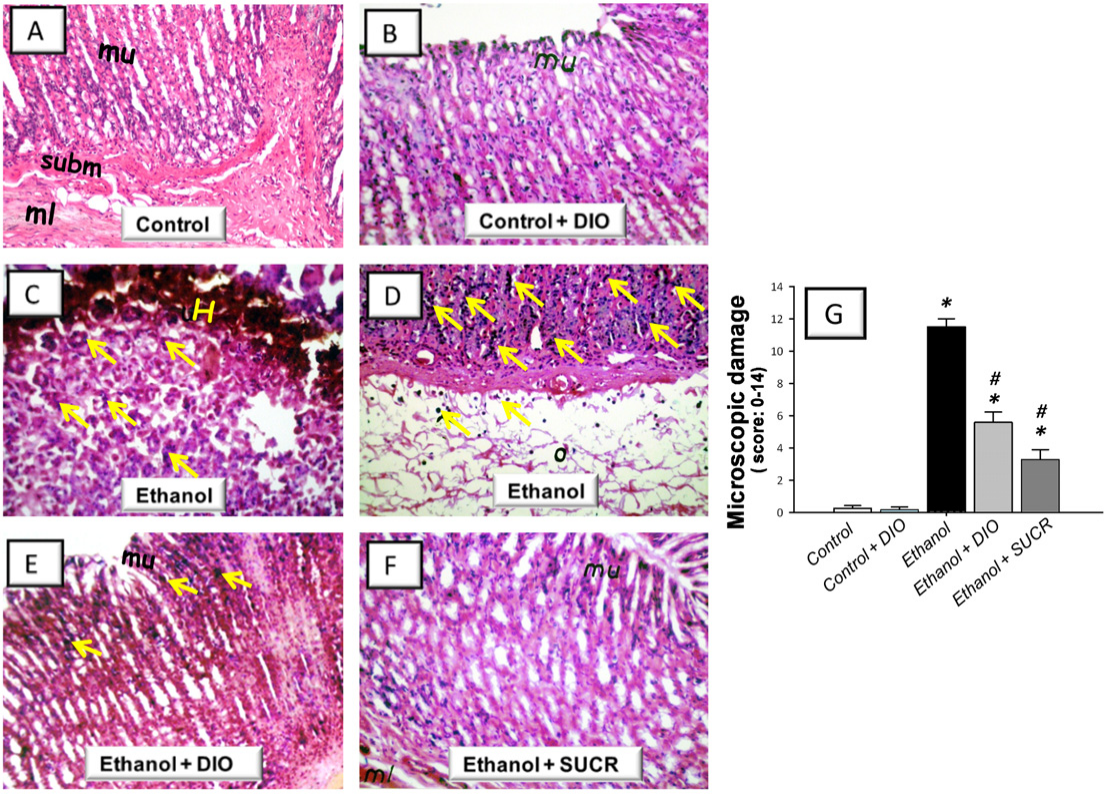
Diosmin alleviates ethanol-induced gastric histopathologic injury in rats. Representative photomicrographs of sections from gastric wall samples taken 1 h post ethanol administration. (A) Control rats receiving saline vehicle showed normal architecture of mucosa (mu) with intact epithelial surface, submucosa (subm) and muscularis (ml) layers. (B) Control rats receiving DIO (100 mg/kg p.o.) elicited no histologic modifications. (C, D) Ethanol-treated group was characterized by mucosal lesions with marked hemorrhage (H).The mucosa was also infiltrated by inflammatory cells (arrows) that also extended to the submucosa which also displayed extensive edema (o). (E) Ethanol + DIO pretreatment (100 mg/kg p.o.) revealed attenuated morphological modifications, diminished inflammatory cell invasion (arrows) and mucosal preservation. (F) Ethanol + SUCR (100 mg/kg p.o.) pretreatment preserved the architecture of the gastric wall. Hematoxylin and eosin staining, original magnification: × 40. (G) Microscopic damage scores (expressed as median; n = 6–8).

The current study next explored whether DIO can protect against the histopathologic damage associated with ethanol-induced gastric injury in rats. Gastric sections from control and control + DIO groups displayed an intact architecture of the gastric wall with few leukocytes in the mucosa (Fig 2A and 2B). In contrast, administration of ethanol triggered a severe gastric injury with high scores of microscopic damage reflecting hemorrhagic necrosis and disruption of the gastric mucosa with epithelial cell loss. This was accompanied with diffuse leukocyte infiltration mainly in the mucosa and submucosa and extensive edema in the submucosal layer (Fig 2C, 2D and 2G). Pretreatment with DIO lowered the pathologic scores signifying the attenuation of gastric damage and inflammatory cell infiltration with preservation of the gastric wall architecture (Fig 2E and 2G). These actions were analogous to those afforded by sucralfate (Fig 2F and 2G).

### Diosmin modulates gastric MPO, inflammatory cytokines and NF-κB p65 protein expression

**Fig 3:**
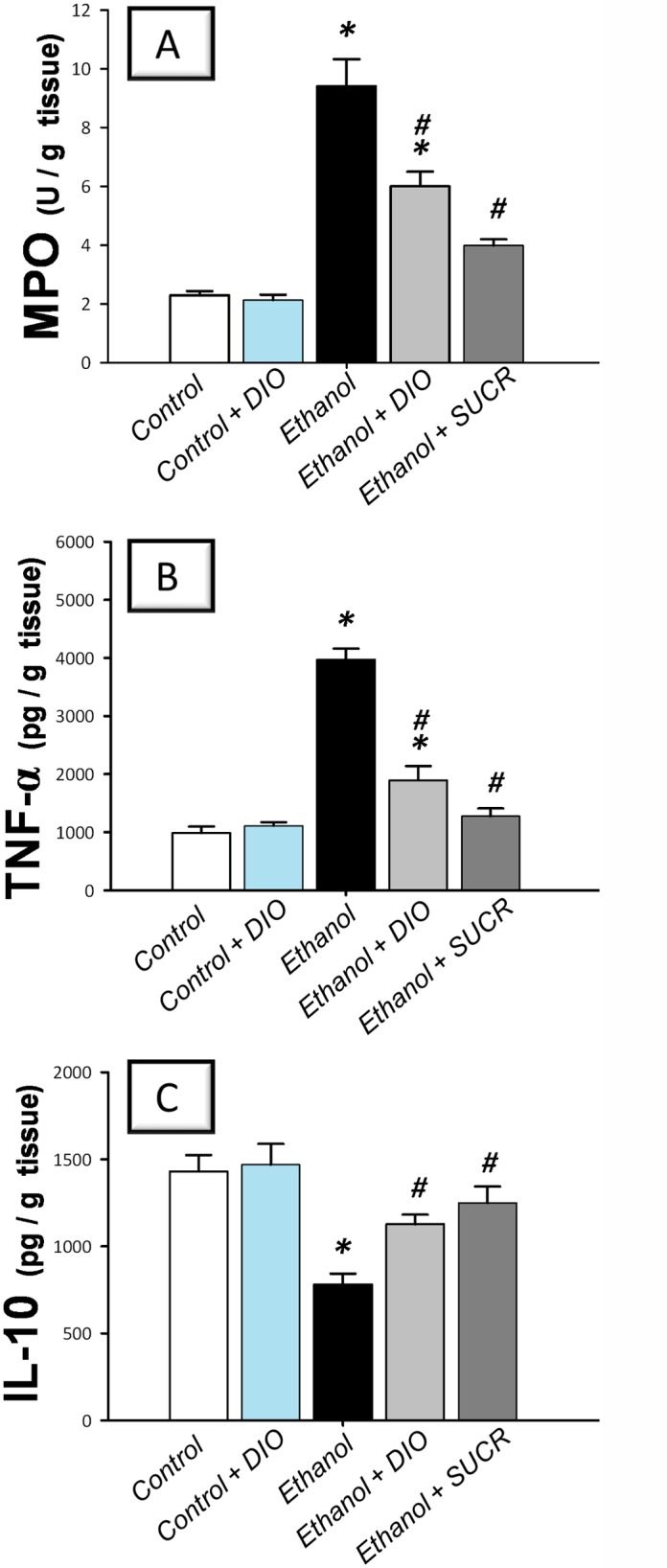
Diosmin attenuates gastric MPO and TNF-α and reinstates IL-10 in rats with ethanol-induced gastric injury. (A) Activity of myeloperoxidase; MPO. (B) Tumor necrosis factor-α; TNF-α. (C) Interleukin 10; IL-10. Measurements were performed 1 h post ethanol instillation and DIO was administered for 1 week before ulcer induction. Data are expressed as mean ± SEM (n = 6–8) *Significant difference from control gp at *p < 0*.*05*, *#* Significant difference from ethanol gp at *p < 0*.*05*. DIO; diosmin, SUCR; sucralfate.

Administration of ethanol resulted in a robust inflammatory response as indicated by a 4.1 fold increase of MPO in addition to a marked elevation of gastric TNF-α (403%) with a concomitant decline of IL-10 (55%) as compared to the control group (Fig 3). Pretreatment with DIO lowered the levels of MPO and TNF-α by 36% and 52%, respectively, and reinstated the IL-10. These favorable actions were similar to sucralfate.

**Fig 4:**
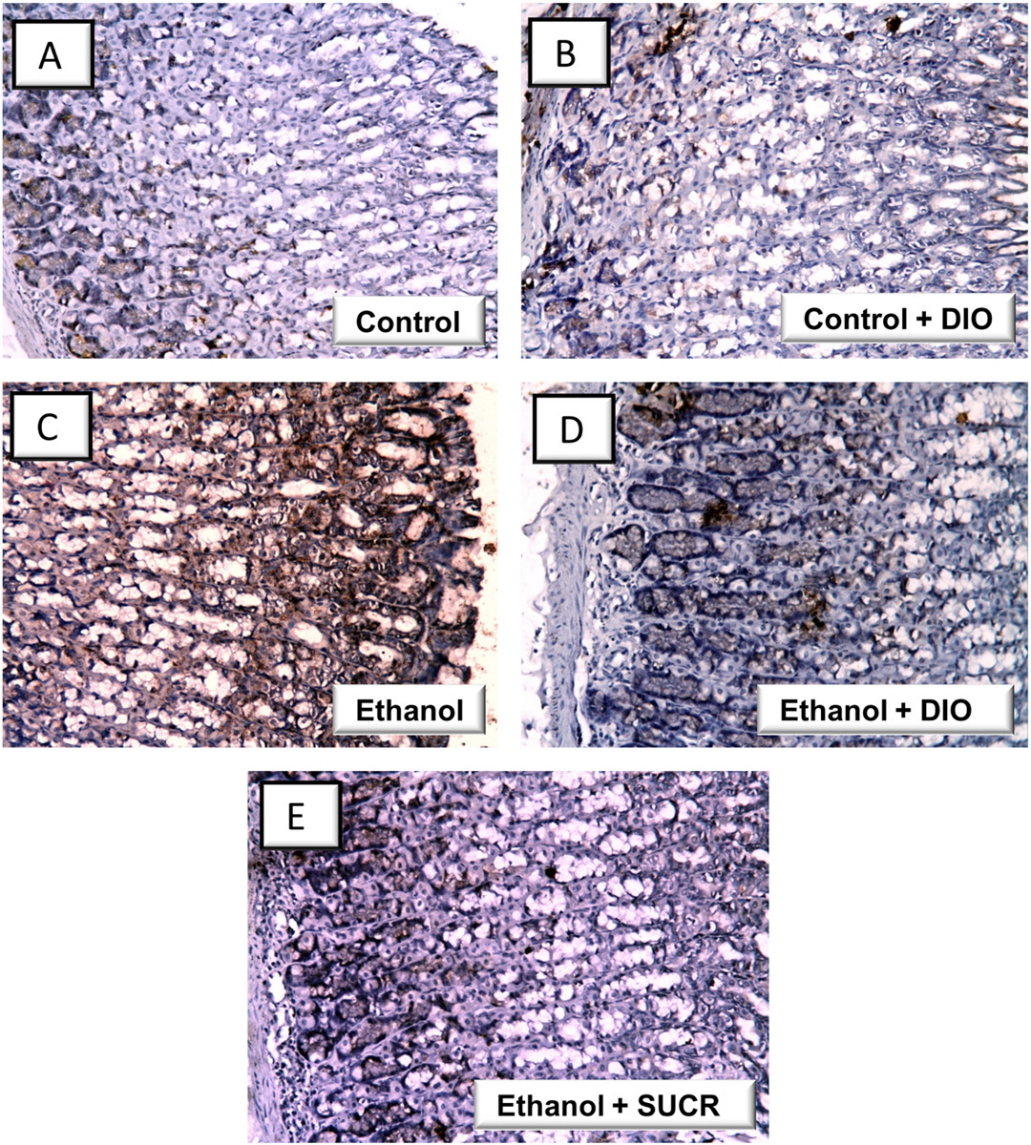
Diosmin downregulates the protein expression of NF-κB p65 in rats with ethanol-induced gastric injury. Representative images for the immunohistochemical detection of activated NF-κB p65 expression from gastric tissues harvested 1 hr post ethanol instillation (magnification: × 200) (A, B) Control and control + DIO gps: minimal expression; (B) Ethanol gp: extensive expression (brown color); (D, E) Ethanol+ DIO and ethanol + SUCR gps: attenuated expression. DIO; diosmin, SUCR; sucralfate.

In the same context, the immunohistochemical detection of activated NF-κB p65 revealed an extensive expression in the gastric tissues of rats treated with ethanol (Fig 4 and Table 1); events which were markedly mitigated by administration of DIO or sucralfate. Together, these observations indicate that DIO modulation of the inflammatory cytokines and NF-κB p65 are implicated in its favorable protective actions against gastric ethanol injury.

**Table 1:** Immunohistochemical detection of NF-κB p65 expression in gastric tissues of rats with ethanol-induced gastric injury.

	Control	Control + DIO	Ethanol	Ethanol + DIO	Ethanol + SUCR
NF-κB p65expression	–	–	+++	+	–

^+++^ extensive,

++ moderate,

^+^ mild,

^—^Nil.

DIO; diosmin, SUCR; sucralfate.

### Diosmin curbs oxidative stress and boosts gastric antioxidant defenses

**Fig 5:**
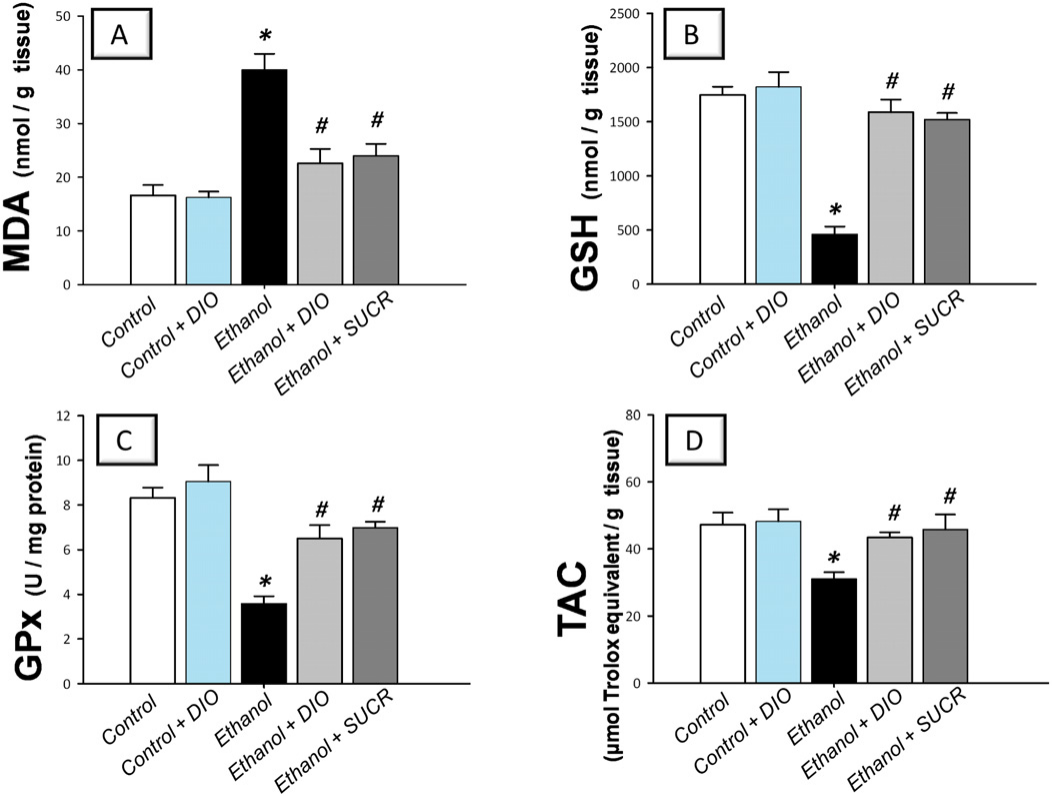
Diosmin lowers gastric MDA and enhances GSH, GPx and TAC in rats with ethanol-induced gastric injury. (A) Lipid peroxides expressed as malondialdehyde; MDA. (B) Reduced glutathione; GSH. (C) Glutathione peroxidase; GPx. (D) Total antioxidant capacity; TAC. Measurements were performed 1 h post ethanol instillation and DIO was administered for 1 week before ulcer induction. Data are expressed as mean ± SEM (n = 6–8) *Significant difference from control gp at *p < 0*.*05*, *#* Significant difference from ethanol gp at *p < 0*.*05*. DIO; diosmin, SUCR; sucralfate.

Instillation of ethanol triggered an oxidative stress as indicated by elevated levels of MDA (240%) with concomitant decline of gastric GSH (26%), GPx activity (43%) and TAC (66%) as compared to the control group (Fig 5). Pretreatment with DIO significantly protected against the oxidative stress as evidenced by lowering of MDA and reinstatement of GSH, GPx and TAC, as compared to ethanol group. These actions were analogous to those afforded by sucralfate. Together, these data suggest that DIO attenuation of oxidative perturbations and enhancement of enzymatic and non-enzymatic antioxidant defenses play a role in mitigation of gastric ethanol injury.

### Diosmin downregulates gastric caspase-3 and Cyt C with restoration of Bcl-2

**Fig 6:**
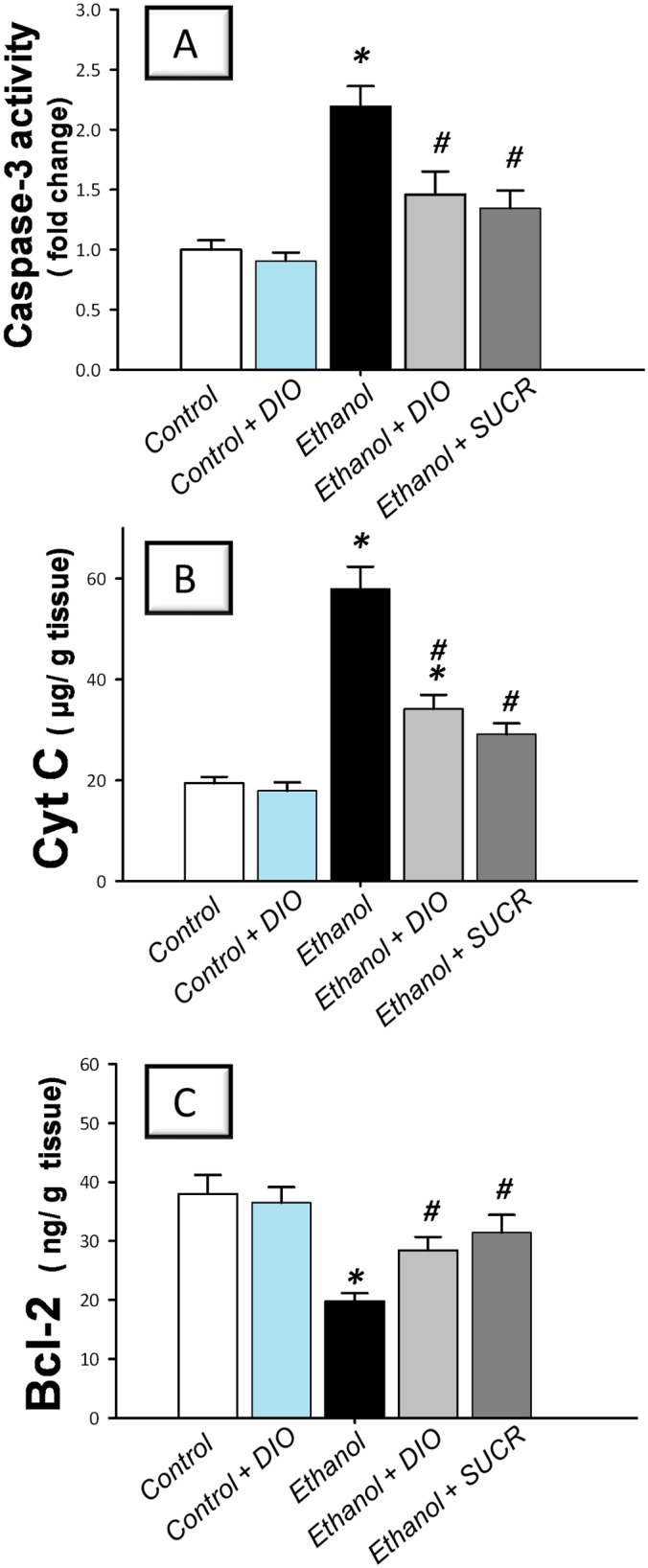
Diosmin suppresses gastric caspase-3 and Cyt C and reinstates Bcl-2 in rats with ethanol-induced gastric injury. (A) Caspase-3 activity. (B) Cytochrome C protein; Cyt C. (C) B cell lymphoma-2 protein; Bcl-2. Measurements were performed 1 h post ethanol instillation and DIO was administered for 1 week before ulcer induction. Data are expressed as mean ± SEM (n = 6–8) *Significant difference from control gp at *p < 0*.*05*, *#* Significant difference from ethanol gp at *p < 0*.*05*. DIO; diosmin, SUCR; sucralfate.

Instillation of ethanol instigated apoptosis in inflamed mucosa as indicated by a 2.4 fold increase of caspase-3 activity, a reliable indicator for apoptosis [3] and a 3 fold elevation of the proapoptotic Cyt C (Fig 6). In addition, the anti-apoptotic Bcl-2 levels were diminished as compared to the control group. Analogous to sucralfate, DIO counteracted these changes in favor of cell survival, implicating suppression of apoptosis as a crucial event in DIO protection against ethanol-induced gastric insult.

### Diosmin replenishes PGE_2_ and NO cytoprotective defenses

**Fig 7:**
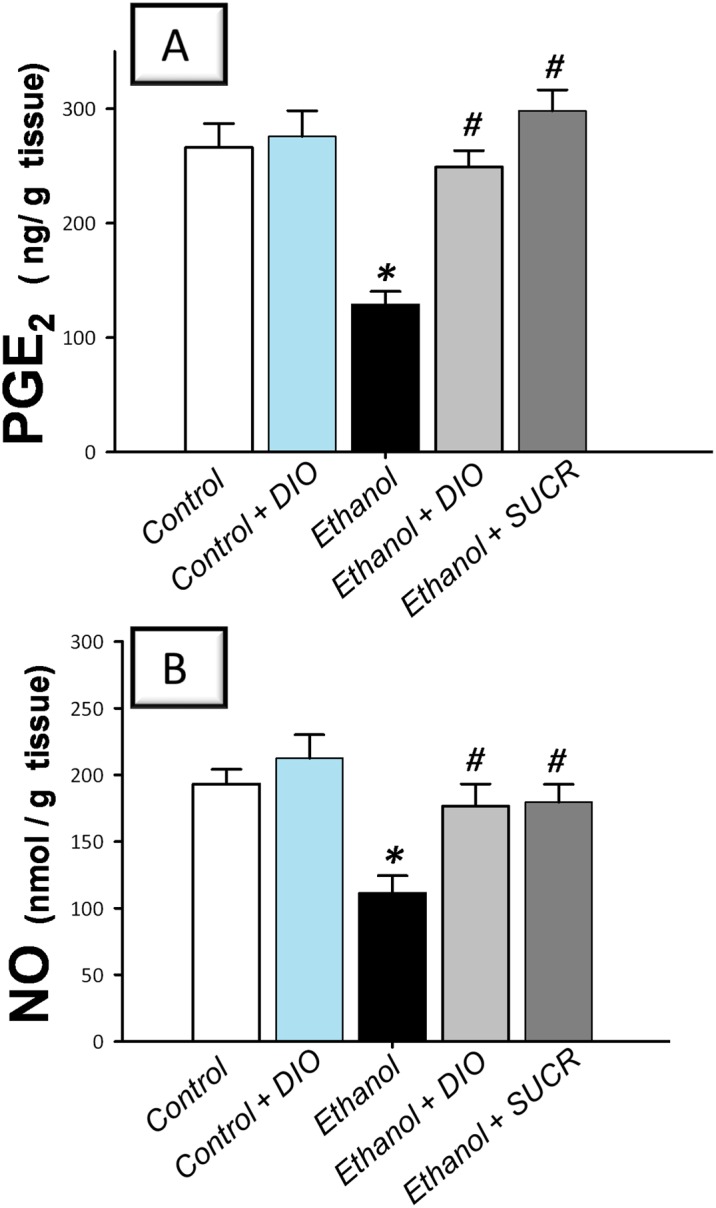
Diosmin boosts the gastroprotective PGE_2_ and NO in rats with ethanol-induced gastric injury. (A) Prostaglandin E_2_; PGE_2_. (B) Nitric oxide; NO. Measurements were performed 1 h post ethanol instillation and DIO was administered for 1 week before ulcer induction. Data are expressed as mean ± SEM (n = 6–8) *Significant difference from control gp at *p < 0*.*05*, *#* Significant difference from ethanol gp at *p < 0*.*05*. DIO; diosmin, SUCR; sucralfate.

Ethanol evoked depletion of PGE_2_ (48%) and NO (58%) levels as compared to the control group (Fig 7). Similar to sucralfate, DIO administration reinstated the levels of these mucosal defenses signifying the role of PGE_2_ and NO enhancement in the attenuation of ethanol injury.

## Discussion

**Fig 8:**
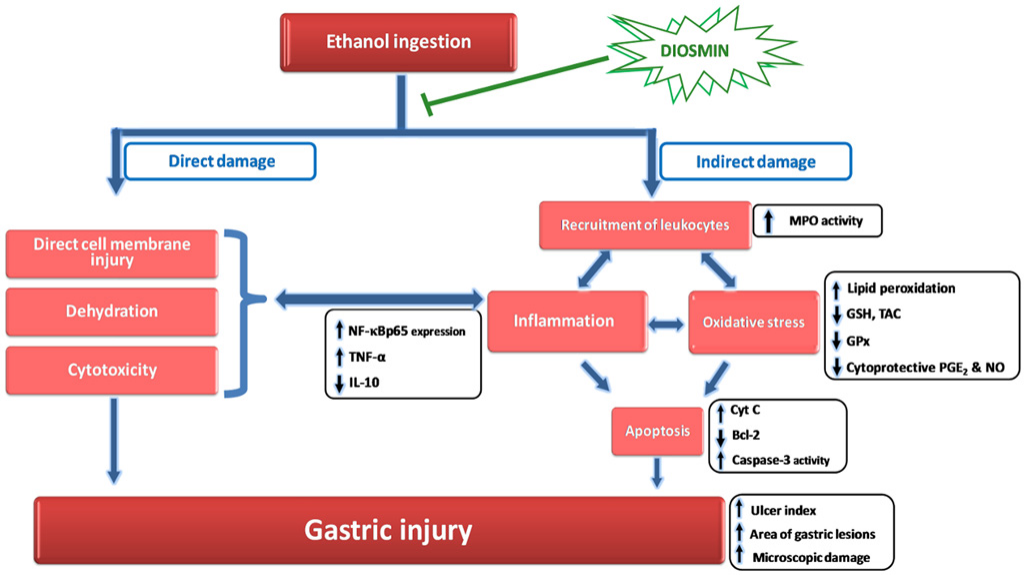
Diagram depicting the ameliorative actions of diosmin in ethanol-induced gastric injury.

The current study highlights, for the first time, the protective actions of DIO, a citrus flavonoid, against ethanol-induced gastric injury in rats. Ethanol inflicts gastric injury via direct effects including disruption of mucosal cellular membranes, dehydration and cytotoxic effects with consequent propagation of the inflammatory cascade [5] (Fig 8). Meanwhile, alcohol causes indirect damaging effects via the recruitment of leukocytes which drives inflammatory responses, oxidative stress and apoptosis. Notably, NF-κB plays a crucial role in mediating the interplay among these events [5, 6]. DIO afforded significant protection against ethanol-induced gastric ulcer mainly through suppression of NF-κB. This was achieved either directly via inhibition of NF-κB downstream targets such as the proinflammatory TNF-α or indirectly via combating ROS by the antioxidant properties of DIO. In addition, the anti-apoptotic and the cytoprotective effects of DIO also mediated the protection against ethanol insult. Generally, these gastroprotective actions were analogous to those exerted by the reference sucralfate signifying the potential use of DIO in alleviating ethanol-provoked gastric lesions.

Ethanol-induced gastric injury is a key experimental model commonly utilized for preclinical assessment of agents with potential anti-ulcer activity since ethanol has been regarded as a leading cause of gastric ulcer in humans [9, 10, 12]. Alcohol has been reported to inflict hemorrhagic gastric lesions characterized by mucosal friability, cellular exfoliation, extensive submucosal edema and inflammatory cell infiltration [5, 40]. In addition, ethanol results in stasis of blood flow and disruption of gastric microvessels; events that inflict hemorrhage and necrotic gastric damage [5, 32]. Injury to gastric mucosa is triggered by invasion of PMN cells as indicated by MPO activity which also generates hypochlorous acid that drives acute inflammation and gastric damage [7]. In the current study, DIO attenuated gastric histopathologic aberrations and leukocyte influx as evidenced by suppression of MPO activity signifying its potential anti-ulcer actions. These observations are in concert with previous studies [5, 32]. Abrogation of neutrophil infiltration has been regarded as a crucial anti-inflammatory mechanism by which effective anti-ulcer agents protect against gastric ulcerative lesions [4]. These favorable actions are likely mediated via the observed DIO inhibition of TNF-α and oxidative stress since they provoke the expression of several adhesion molecules, including ICAM-1, that enhance leukocyte invasion to injured gastric mucosa [5, 41].

The current data revealed that ethanol ingestion upregulated the inflammatory response as evidenced by increase of gastric proinflammatory TNF-α and enhancement of the protein expression of activated NF-κB p65 in rats. This was accompanied with a decline of the anti-inflammatory IL-10. These findings are consistent with previous reports [4, 9, 32]. TNF-α has been tightly linked to gastric inflammation via activation and recruitment of immune cells, generation of other proinflammatory cytokines and upregulation of NF-κB [32, 42]. TNF-α also suppresses gastric microcirculation around ulcerated mucosa and thus delays its healing [43]. Conversely, IL-10 has been reported to downregulate MHC class II antigen presentation and subsequent release of pro-inflammatory cytokines and thus, its diminished levels exacerbate gastric lesions [4, 9]. In the same context, NF-κB is a transcription factor that mediates crucial inflammatory events in ethanol-induced gastric injury including the expression of several downstream proinflammatory targets such as TNF-α, chemokines as IL-8 and adhesion molecules [6, 32, 44, 45]. In the inactive state, NF-κB, a heterodimer of p65 and p50 subunits of the Rel protein family, is located in the cytosol of cells and is kept inactive via binding to the inhibitory protein IκBα. Upon exposure to stress signals such as ROS and inflammatory cytokines, the IκBα undergoes phosphorylation and subsequent proteasomal degradation. As a result, activated NF-κB is released which then translocates to the nucleus to interact with DNA response elements to mediate the transcription of target inflammatory genes [46]. Thus, NF-κB p65 subunit has been commonly regarded as a marker for NF-κB activation [32, 47, 48]. Notably, several inflammatory mediators have been identified to activate the NF-κB such as TNF-α and IL-1β which generate ROS, thus implicating ROS as a common activator of NF-κB [45].

Interestingly, DIO suppressed gastric MPO, TNF-α and NF-κB p65 with enhancement of the anti-inflammatory IL-10 levels. It can be presumed that the inhibition of NF-κB is a chief mechanism for DIO suppression of gastric inflammatory response since the expression of several proinflammatory cytokines including TNF-α is mainly regulated by the transcription of NF-κB [32, 42]. Thus, the inhibition of NF-κB p65 expression together with its downstream targets such as MPO and TNF-α has been viewed as a successful strategy for the management of gastric injury [19, 32]. Meanwhile, the above findings reinforced the histopathologic results that described DIO attenuation of immunoinflammatory cell infiltration, edema and hemorrhage. Previous reports revealed that the marked anti-inflammatory actions of DIO mediated its protection against hepatic injury [19] and hepatocarcinogenesis [20] via suppression of TNF-α and NF-κB. Thus, the current data demonstrate the protective actions of DIO in ethanol-induced gastric damage, which were partly mediated via its multi-pronged immunomodulatory and anti-inflammatory actions.

The involvement of oxidative stress in the pathogenesis of ethanol-induced gastric injury has been confirmed by several studies. In this regard, the surge of ROS generated by activated neutrophils and macrophages along with hypochlorous acid synthesized by MPO trigger gastric mucosal injury [5, 9, 49]. Ethanol has been reported to cut gastric mucosal microcirculation resulting in hypoxia, ROS generation and associated lipid peroxidation [41]. In the present study, ethanol administration instigated gastric oxidative stress and increased the levels of lipid peroxides in a process driven by neutrophil activation. It also depleted the gastric GSH, GPx and TAC antioxidant defenses which scavenge free radicals and prevent their detrimental effects [5]. Depletion of GSH, which plays a central role in combating oxidative stress and cellular damage, renders gastric tissues more vulnerable to oxidative injury [5, 50]. In addition, suppression of GPx, a vital antioxidant defense which guards against oxidative aberrations, exacerbates ethanol-evoked gastric injury [2].

Data of the current study demonstrated that DIO combated oxidative stress and enhanced the antioxidant status in animals with ethanol gastritis as revealed by reduction of MDA levels in addition to augmentation of GSH, GPx and TAC. These findings are in agreement with previous studies and they highlight the premise that the antioxidant features of DIO are implicated in the alleviation of ethanol gastric injury [15, 17, 18]. The antioxidant actions of DIO have been ascribed to scavenging hydroxyl radicals and superoxide anions [17]. An additional plausible explanation for the antioxidant effects of DIO can be attributed to the abrogation of neutrophil infiltration observed in the current study. In fact, scavenging of ROS has been regarded as one of the mechanisms implicated in healing of ulcers [4]. The antioxidant actions of DIO can play a role in attenuation of gastric inflammatory response via inhibition of the redox-sensitive NF-κB cascade [6]. In addition, the preservation of GSH, GPx and TAC defenses signifies the role of DIO in boosting the mucosal antioxidant defenses and correlates well with the reported preservation of endogenous antioxidants in experimental hepatic [19] and renal [15] injuries along with diabetes mellitus [18]. Together, the observed antioxidant actions likely contribute to the protection of DIO against mucosal injury.

The present results also described an *in vivo* activation of apoptosis in ethanol-treated gastric tissues as demonstrated by upregulation of Cyt C and caspase-3 with decline of Bcl-2 levels. These data are in concert with previous studies [2, 3, 14]. Enhanced apoptotic death of gastric epithelial cells has been partly implicated in ethanol-induced gastric mucosal injury [2, 51, 52]. Inflammatory signals along with oxidative stress have been reported to instigate the expression of several genes responsible for cellular death by apoptosis [4, 53]. Apoptotic cascade is initiated by pro-apoptotic signals such as Bax [2, 29] which favor the release of Cyt C from the mitochondria to the cytosol, with subsequent activation of caspase-9 and ultimately caspase-3, the major executioner caspase [3, 14].

The current data revealed that DIO suppressed Cyt C and caspase-3 and augmented the anti-apoptotic Bcl-2, indicating attenuation of gastric mucosal apoptosis. These findings are consistent with previous reports that described the inhibitory effects of DIO against apoptosis in experimental renal injury via downregulation of p53, Bax and caspase-3 expression [15]. The attenuation of mucosal apoptosis can be ascribed to the observed suppression of lipid peroxidation and TNF-α since excessive exposure of gastric mucosa to ROS and TNF-α has been reported to enhance gastric epithelial apoptosis [2, 4, 29]. Additionally, the observed DIO boosting of PGE_2_ may be partly implicated in apoptosis suppression since PGE_2_ has been reported to enhance the expression of Bcl-2 [54].

The present data also indicated that ethanol ingestion lowered the levels of PGE_2_ and NO cytoprotective moieties; findings that coincide with previous studies [9, 11]. The interplay among gastric PGE_2_, NO and GSH has been implicated in maintaining the viscoelastic layer of mucus that plays crucial roles in protecting the underlying mucosa from aggressive factors [22, 55]. Gastric PGE_2_ as well as NO augment mucosal defenses via boosting of mucus and bicarbonate secretion, maintenance of mucosal blood flow and abrogation of leukocyte infiltration [22, 56, 57]. In addition, PGE_2_ suppresses the surge of proinflammatory mediators including histamine, TNF-α and IL-1 from macrophages [58]. NO has been reported to upregulate PGE_2_ biosynthesis via cGMP-independent mechanisms [59]. Meanwhile, GSH augments prostaglandin action and stabilizes mucus composition by controlling the thiol/disulfide ratio [22]. In the current study, DIO enhanced the levels of PGE_2_, GSH and NO mucosal defenses, signifying the contribution of these targets to the alleviation of ethanol mucosal insult. Meanwhile, the observed boosting of NO could be ascribed to DIO quenching of the superoxide anion which consumes NO for the generation of the cytotoxic peroxynitrite [60].

In the current study, the observed beneficial actions of DIO were analogous to those exerted by sucralfate, the standard anti-ulcer agent. Sucralfate has been reported to protect the gastric mucosa against noxious irritants and accelerate the healing of chronic ulcers [27]. Its marked antioxidant features abrogate lipid peroxidation and preserve gastric antioxidant defenses [24]. It also acts by boosting of mucus secretion and NO biosynthesis [25, 28]. Meanwhile, sucralfate suppression of the proinflammatory cytokines adds to its efficacy as an antiulcer agent [25].

## Conclusions

In conclusion, the current study highlights evidences for the protective effects of DIO in a rat model of ethanol-induced gastric ulcer. These favorable actions were mediated via suppression of gastric inflammation and oxidative stress, chiefly through NF-κB inhibition. Meanwhile, curbing of apoptosis and boosting of mucosal antioxidant and cytoprotective defenses substantially contributed to DIO protection against ethanol damage. Interestingly, the beneficial effects of DIO were similar to those exerted by the reference antiulcer sucralfate. Thus, the current study pinpoints to the benefits of DIO as an effective and safer approach for the management of gastric ulcers. Further studies are warranted to investigate the potential efficacy of DIO in the clinical setting as an adjunct approach for the management of gastric ulcers. In addition, the detailed molecular mechanisms of DIO and the implicated signaling networks including NF-κB signaling need to be explored.
